# The Perceptions of International Learners Toward Teacher Code-Switching in the Elementary and Intermediate Chinese Foreign Language Classrooms

**DOI:** 10.3389/fpsyg.2022.860567

**Published:** 2022-04-04

**Authors:** Yuqi Hu, Muhammad Afzaal, Hind Alfadda

**Affiliations:** ^1^Department of Chinese and Bilingual Studies (CBS), The Hong Kong Polytechnic University, Kowloon, Hong Kong SAR, China; ^2^Institute of Corpus Studies and Applications, Shanghai International Studies University, Shanghai, China; ^3^College of Education, King Saud University, Riyadh, Saudi Arabia

**Keywords:** code switching < language learners, student perceptions, CFL classrooms, education - active learning, langauge acquisition

## Abstract

The present study explores the perceptions of elementary and intermediate learners toward English as a code-switching medium in Chinese as a Foreign Language (CFL) classrooms. The data were collected by means of a survey of international elementary and intermediate-level students studying Chinese at the College of Chinese Language and Culture (CCLC) of Jinan University in Guangzhou, China. The data collection method included a five-point Likert scale questionnaire survey (18 closed-ended questions and one open-ended question). The analysis of quantitative and qualitative data collected from the questionnaire showed that CFL learners at both proficiency levels tended to favor code-switching in classrooms. However, the results of the Mann–Whitney U test showed that intermediate learners favored code-switching to a lesser extent than elementary learners. Moreover, their perceptions were found to differ significantly in terms of the varying functions of code-switching. These findings suggest that code-switching functions as an effective teaching strategy in the CFL classrooms, although teachers should regulate its deployment when teaching learners with different proficiency levels.

## Introduction

In recent decades, the role of code-switching in teaching foreign languages has received widespread global attention. In Chinese as foreign language (CFL) classes, code-switching is often employed to fulfill a variety of conversational and educational purposes in China.

Code-switching is viewed as a key resource for effective bilingual communication because it enables students to use their bilingual competencies in class as they do outside, thus conceptualizing the classroom as a bilingual space in which participants mirror interactional patterns found in situations outside the classroom (Liebscher and Dailey-O'cain, 2005). According to this viewpoint, the language classroom is viewed as a community of practice (CoP) with shared norms and understandings that allow the students and the instructor(s) who are participants in the CoP to achieve a common educational goal and boost professional development by using the languages available to them (Al-Habsi et al., 2022, p. 51).

CFL is a field gaining increasing attention and importance in the current society. The current study is conducted within a CFL context. Both teaching Chinese as a foreign and as a second language are the corresponding translation of the original Chinese term 对外汉语教学, pinyin: duìwài hànyŭ jiàoxué, literarally means “Chinese language teaching towards outside” (Li and Wang, 2016), which is a traditional label for the academic discipline ever since 1982 in China, referring to Chinese teaching and learning activities in China (Cui, 2010). However, the two terms refer to different situation. According to Wang (2010), CSL is regarded as either teaching Chinese as a second language to minority groups in China, Taiwan, Singapore, and Hong Kong, or as a “heritage and community language in the diasporas across the world” (p. 252–253) such as the United States and Britain. On the other hand, CFL targets international learners with multiple native languages within China. Even though English is the world’s most widely spoken language, the number of international students choosing to study in China has expanded substantially. The total number of overseas students in China has increased by approximately 10% annually over the last 2 years (Ministry of Education of the PRC, 2018). When it comes to overseas students studying in Chinese higher education institutions, 49.38 per cent (241,500) of those who enrolled in institutions within China in 2017 joined degree programs (Ministry of Education of the P.R.C, 2018). The bid to join the global race for hosting overseas students has been confirmed by Chinese authorities.

Moreover, the study of Chinese appears to be drawing wider interest worldwide with the momentum driven in part by the support of the Office of Chinese Language Council International (also known as Hanban) and the Confucius Institute program (Zhao and Huang, 2010; Osborne et al., 2018). By the end of 2017, 525 Confucius Institutes had been established in 146 countries (Hanban, 2018). It has been estimated that by the end of 2023 approximately 1.1 million people will have enrolled in Confucius Institute programs worldwide (Yuan and Zheng, 2014). Furthermore, an estimated 100 million people may be studying Chinese in schools, universities, and private institutions (Wu, 2007).

For many years, the Chinese-only norm in TCFL was adhered to in practice and in academic discourse. This stance could be attributed to a belief by the educators that the monolingual environment increases students’ exposure to the target language and thus facilitate their language errors, allowing instructors to make corrections in time (Lin, 2013). The monolingual norm is also due to the unproven assumption that Chinese is best taught through the target language (Wang, 2019). However, it is also pointed out that teachers and students generally perform code-switching between Chinese and English in CFL classrooms (Wang, 2010, 2014). Indeed, the active involvement of teachers’ and students’ linguistic repertoires in the perceived code-switching reality is viewed as challenging the target language as the only acceptable mode of communication.

Even though the teaching and learning of Chinese as a foreign language in China and abroad has attracted considerable scholarly interest, this area still needs the attention of researchers, scholars, policymakers, and teachers. In this regard, the present study addresses the gaps in existing literature by examining the perceptions of CFL students toward the code-switching practice in class.

## Literature Review

Fromkin and Rodman (1998) define code-switching as the “insertion of a word or phrase from a language other than that being spoken into a single sentence, or the switching back and forth between two languages or dialects” (Fromkin and Rodman, 1998, p. 522). Expanding the definition offered by Fromkin and Rodman to include interlocutors, Cook (2000) describes code-switching as “changing from one language to another in the middle of a speech when both speakers are conversant in the same two languages” (Cook, 2000, p. 83).

Research on code-switching has generally examined the switches between languages at lexical or sentential levels (e.g., Ye, 2021; Caballero and Celaya, 2022), while translanguaging is defined to include alterations beyond the scope of the sentence or even discourse level (*cf*, Williams, 2000; García, 2009). According to Goodman and Tastanbek (2021), translanguaging involves language switches between teaching and learning modes, which engages the participation of both the teacher and the students. The terminology of code-switching is deemed more suitable to be adopted in the current study, since it focuses on the teacher’s momentary switch between English and Chinese language. This research scope is determined by the classroom norms in CFL programs in China: the “dominant monolingual Chinese-only policy” and traditional “teacher-centered” pedagogy (Wang, 2014, 2019), which constraint the frequency and modes of teacher’s and students’ usage of translanguaging.

Within this context, the present study focuses on the discourse functions as instances and interactional strategies (Gumperz, 1982) in which code-switching is used to sustain the ongoing conversation in the classroom context for discourse continuity. A number of scholars have explored the functions and rationales of teachers’ code-switching practices in language teaching classrooms. For instance, Martin (1999) proposes seven reasons why language teachers code-switch in language classrooms. These range from code-switching being used to signal the transition from preparing for a lesson to starting the lesson, specifying a specific addressee, distinguishing “doing a lesson” from “talking about the lesson,” changing footing or making an aside, distinguishing questions from a written text and foregrounding the voices of different characters in a narrative and to entailing classroom management.

Arguing that making use of the L1 in L2 classrooms is humanistic, Cook (2000) observes that the learners’ ability to express themselves is not hampered by a lack of vocabulary or a fear of making mistakes when using this approach. Cook suggests that rather than viewing code-switching as a hindrance, teachers should view it as a means to facilitate and ease the learning process. He proposed that teachers’ code-switching is likely to be most effective for grammatical explanations task organization, student disciplining, and test administration.

According to Greggio and Gil (2007), teachers tend to code-switch with the beginner group to provide grammatical explanations, give instructions, monitor/assist the students, correct activities, and to attract their learners’ attention. In addition, the researchers found that most teachers report that they needed to code-switch to “clarify words, expressions, structures, and rules of utterances” (Greggio and Gil, 2007, p. 376). Thus, based on the functions elaborated above, code-switching plays an important role in the ESL classrooms by assisting students in better understanding the target language they are learning.

Other researchers such as Ahmad and Jusoff (2009) highlighted that code-switching provided students with opportunities to communicate and improved students’ understanding. In addition to facilitating classroom instruction, they observed that CS promoted adequate information and skill transfer (Ahmad and Jusoff, 2009) which leads to a better understanding of learning among learners because it provides them with enough input to understand the L2.

Previous studies have also looked into students’ attitudes toward teachers’ code-switching. For example, Duff and Polio (1990) discovered that students were satisfied with their teachers’ language choices, even though the use of L1 ranged from 0 to 90%. Macaro (1997) investigated L2 exclusivity and adult learners’ attitudes, revealing that students reported being either demotivated when faced with L2 exclusivity or simply decided to “go along with it” (p. 127). Investigating both students’ and teachers’ attitudes toward, Levine (2003) investigated learner anxiety when confronted with target language use. According to Levine, while some students felt anxious when exposed to the target language, it was beneficial to use it in teaching rather than to rely on the students’ first language. The findings of these studies appeared to be mixed, with students sharing that the effectiveness of L1 use rather than the quantity of teachers’ L1 use played a significant role in their satisfaction.

The results of other studies on code-switching demonstrate how teachers and students perceive and react to code-switching behaviors in the classroom. For example, according to the findings of the study carried out by Ibrahim et al. (2013), code-switching in the classroom was evaluated positively by teachers because it was used for educational goals. A study conducted by Rivera and Mazak (2017) indicated that students had neutral to favorable attitudes on code-switching. As a result, the students frequently used code-switching in the classroom. Songxaba et al. (2017) found that most teachers who participated in their study considered that code-switching was the most effective method for assisting students in understanding the lectures given by their professors. According to the findings of the study by Songxaba et al. (2017), code-switching is suitable for lower-level classes but should be discouraged in higher-level classes.

Scholars also have argued that the practice of code-switching is included in translanguaging (*cf*, García, 2009; Sayer, 2013). As a result, the researchers also revisit the literatures of translanguaging so as to conduct a tentatively exhaustive review. Zhang et al. (2020) adopted the method of interviews and classroom observations to investigate the teachers’ and students’ practice and perceptions of translanguaging (code-switching) in five Chinese universities. The scholars presented their data by categorizing the practice of translanguaging into teacher’s pedagogical translanguaging (offering classroom instructions; managing classroom discipline; introducing grammatical or lexical items; checking students’ understanding of information; and correcting pronunciation) and students’ spontaneous translanguaging (asking and answering questions from the teacher; asking and answering questions from classmates a question). Danping Wang (2020) employed a mixed-method approach which integrated questionnaires and interviews to investigate translanguaging reality in three Mandarin Chinese programs at New Zealand universities. Contrary to the requirement of the monolingual principle, it was found that CFL students at higher education frequently employed code-switching in the classrooms, as they regarded it as an effective tool to scaffold knowledge and relieve stress. Zheng (2020) adopted an ecological perspective to analyze interactional discourses which emerge from teacher’s and students’ translanguaging practice in a K-12 Chinese immersion classroom in the United States. It was observed that translanguaging strategies through various modalities were effective, including the employment of English, Chinese characters, pinyin, icons, and colors. The research also argued that students’ practice of code-switching is influenced by the locally negotiated social norms and context and gives relevant pedagogical implications.

The literature review presented above has showed that various studies have been conducted to investigate students’ perceptions toward code-switching in the EFL context; however, few studies focusing the similar topic have been done within the CFL context. Danping Wang has done a series of studies exploring the teaching and learning of the Chinese language internationally. She has explored CFL learners’ attitudes toward the use of English as a *lingua franca* (ELF) and the Chinese-only pedagogy in her 2010 article and 2014 book. These studies have showed the existence of discrepancies between theory and practice of the usage of instructional medium in CFL classrooms. Such foundational findings suggest that students favor the ELF pedagogy to be adopted by teachers to aid their more effective learning of Chinese. However, further investigations remain to be done in terms of how code-switching, a specific way to realize the ELF pedagogy, supports their learning and in what aspects. Wang and Kirkpatrick’s (2012) article investigates CFL teachers’ beliefs of switching to English as code in CFL classrooms. Their 2019 study explores such perceptions of both teachers and students, with only the beginner-level students being included. Wang also covers the area of CSL (see Wang, 2016, 2020). The gap thus lies in the exploration of CFL students’ perceptions toward English as a medium of code-switching and the comparison of their attitudes across different proficiency levels. The current study aims to fulfill such vacancy. As the China has been attracting increasing amount of people working and living within the country due to the Belt and Road Initiative (BRI, 一带一路), it is of significance to investigate and boost the understandings toward the field of CFL pedagogies.

Therefore, the present research sought to address this gap by inquiring into students’ attitudes toward code-switching in CFL classrooms and ascertaining if their opinions varied across their Chinese language proficiency levels.

To address the above-stated problems and gaps, three research questions were formulated as:

1. What are the students’ overall perceptions of teachers’ code-switching in Chinese as a foreign language (CFL) classroom?
2. According to students with different proficiency levels, when does code-switching best function in CFL classrooms?
3. Are there any significant differences in students’ perceptions contingent upon their different Chinese-proficiency levels?

## Methodology

The present research adopted a mixed-methods approach to investigate the perceptions of CFL learners across different proficiency levels toward code-switching. The quantitative data included those gathered from the 18 closed-ended questions in the questionnaire. The qualitative analysis involved the analysis of the responses to the open-ended item in the survey, which served as a supplement to the statistical analysis.

### Setting and Participants

The research setting is the College of Chinese Language and Culture (CCLC), Jinan University, Guangzhou, China. According to the school official website,^1^ the Department of Chinese Language enrolls international students who have the intentions to learn Mandarin Chinese language and equip themselves with abilities to work in the context of inter-cultural communication. It enrolls over 2,000 international students from the United States, Canada, the United Kingdom, Australia, Korea, Japan, Southeast Asia countries, and Africa. English and Chinese are the medium languages for classroom teaching and instruction and daily communication. The college offers Chinese language courses at various levels, including the elementary, the intermediate, and the advanced levels.

The sample of 56 students was selected from two different Chinese-proficiency-level classes, namely, the elementary- and the intermediate-level class. The students from CCLC are disseminated into different classes when they started learning at CCLC. The students who had secured scores ranging between 40 and 69 are categorized into the elementary class, while those who had attained scores between 70 and 100 in the test belonged to the intermediate class. The participants selected in the current study included 28 elementary-class students and 28 intermediate-class students. Altogether, 22 valid responses were collected from the elementary class, and 21 valid responses were obtained from the intermediate class. To ensure that an equal number of questionnaires across the class levels were analyzed, 21 out of 22 sheets were selected randomly from the data collected from the elementary class. In total, 42 sheets were subjected to analysis. The students were adult aged between 18 and 55, with 25 of them being males and 17 females. They were from 16 countries and spoke 12 different languages, with Thai (*N* = 15), Russian (*N* = 7), Arabic (*N* = 4), and Indonesian (*N* = 4) being the four languages spoken the most as their native language. Among these learners, 37 of them reported that they spoke English other than their L1 and Chinese, 2 of them spoke English as their L1, while 3 only spoke their L1.

### Research Instrument

The questionnaire used in the present study featured three sections, comprising demographic information, 18 closed-ended questions, and one open-ended question. The closed-ended questions are categorized into four categories: overall attitude, subject access, classroom management, and interpersonal relations.

The demographic part required the students to fill in some basic information such as their gender, years of learning Chinese, and age range. The students were not required to share their names in this survey. Such identifying details were avoided to ensure that the students were able to express their perceptions and views freely without any apprehension as to the possibility of their opinions being accessible to those who were teaching them.

The closed-ended questions contained three items from the survey used by Nordin et al. (2013, p. 482–484) and 15 items from Yao’s questionnaire (2011, p. 20–24). All questions required the subjects to measure a statement based on a 5-point Likert scale as follows: Strongly Agree = 5; Agree = 4; Not Sure = 3; Disagree = 2; and Strongly Disagree = 1. The first section was a modification from Nordin et al.’s (2013) survey, which was aimed at eliciting the students’ general perceptions toward teacher’s code-switching. The other three categories were modified from Yao (2011), with each section focusing on participants’ attitudes to functions of teacher’s code-switching in terms of three areas, namely, subject access, classroom management, and interpersonal relations. The teacher’s persona in Yao’s original questionnaire (*ibid*) was excluded from the present survey as this set of questions was used to address both teachers’ and students’ attitudes.

The single open-ended question was included to elicit more detailed and specific views from the students and to provide complementary or explanatory information for the data extracted from the closed-ended questions. The open-ended question is situated at the end of the questionnaire. After ticking the 18 items contained in the closed-ended part, the students were invited to write down their comments on the question—“Do you feel good or bad when teachers use English in class? Why?”

The questionnaire was delivered in English, since 93% of the participants spoke English as their L2 or L1 (see section “Setting and Participants”). Two approaches were conducted to secure the participants’ comprehension of the questionnaire items. First, the teacher who were teaching the concerned elementary and intermediate classes at the time of the survey was invited to assist the students to understand the basic meanings of the questionnaire. She was experienced and was familiar with the students, and thus was able to give instructions and explanations to the students with high frequency and simple Chinese expressions. Second, some modifications on the previous questionnaires included substituting the expressions of “code-switching” and “(teachers) who use code-switching” with “use of English” and “(teachers) who switch from Chinese to English,” respectively. In addition, the academic terminology was simplified with equivalent meaning so that it was easier for the students to understand and avoided confusion. Some expressions which were too academic were changed to plainer language, such as “enliven the atmosphere” to “make the atmosphere relaxing,” “negotiate with students,” and to “make the students feel closer to the teachers.”

### Data Collection and Analysis

The specific data collection processes are as follows: First, the researcher secured informed consent from the class teacher and students to participate in the survey. Then, during recess time in both classes, the questionnaire was administered to the students for 10 min. Prior to responding to the survey, the students were furnished with the research purpose and a verbal explanation of code-switching by the researcher in English. Then, the class teacher explained the basic meanings of the questionnaire items to the students. During the time the students responded the survey, they were allowed to ask either the teacher or the researcher freely if encountered with any problems.

The analysis of the data in the study comprised three steps. Firstly, frequencies of students’ responses to the 18 closed-ended questions were keyed into Excel for examining the proportions of their choices. This step enabled the explication of the students’ overall perceptions and attitudes toward code-switching in addition to its three functions, thus addressing research questions 1 and 2. Research question 3 was addressed through the remaining two steps. The five-point Likert scale data were imported into SPSS to run the Mann–Whitney U test for comparing the means of the two sets of data and checking for significant divergences, if any, between the two groups in terms of different functional areas of code-switching. Since the data were ordinal with two groups (elementary and intermediate) of the independent variable, the nonparametric Mann–Whitney U test was employed. Finally, the contents collected from the open-ended questions were analyzed to inquire into any elaborations on and explanations of their perceptions. Of the 42 valid questionnaires, 28 sheets are left with comments by the student participants. However, only 25 comments were valid, since the remaining 3 sheets, the information provided by the students were not relevant. For instance, one student commented on the quality of the teacher instead of her code-switching practice: “teacher Yuan is very nice.” Additionally, 4 students opted to write their comments in Chinese, which were translated by the researchers into English later. In total, the 42 participants were assigned the labels S1 to S42. S1 to S21 comprised elementary-level students, while S22 to S42 comprised intermediate-level students.

## Results and Findings

The Cronbach Alpha is a measurement of internal consistency which is suitable for assessing Likert scale items (Cronbach, 1951). The values thus indicate the consistency levels of the items in questionnaires, with the value of 0.70 or above being commonly regarded as “acceptable” in most social science research. The Cronbach Alpha values for the present study were generated by SPSS (Table 1). The Cronbach Alpha values for the elementary and intermediate groups are 0.938 and 0.946, respectively, suggesting that the reliability is high for the answers of both groups. The Cronbach Alpha values for the four sections of the elementary students are 0.612, 0.828, 0.874, and 0.924, and the values for the four sections of the intermediate students are 0.724, 0.918, 0.893, and 0.805, respectively. The data thus show that the items in the current questionnaire adopted are consistent.

**Table 1:** The Cronbach alpha values for the elementary and intermediate students.

The cronbach alpha’s values	Overall perception (18 Q^1^)	The general perception (Q 1–3)	Subject access (Q 4–8)	Classroom management (Q 9–13)	Interpersonal relations (Q 14–18)
Elementary students	0.938	0.612	0.828	0.874	0.924
Intermediate students	0.946	0.724	0.918	0.893	0.805

1 *Q: Questions*.

**Figure 1:**
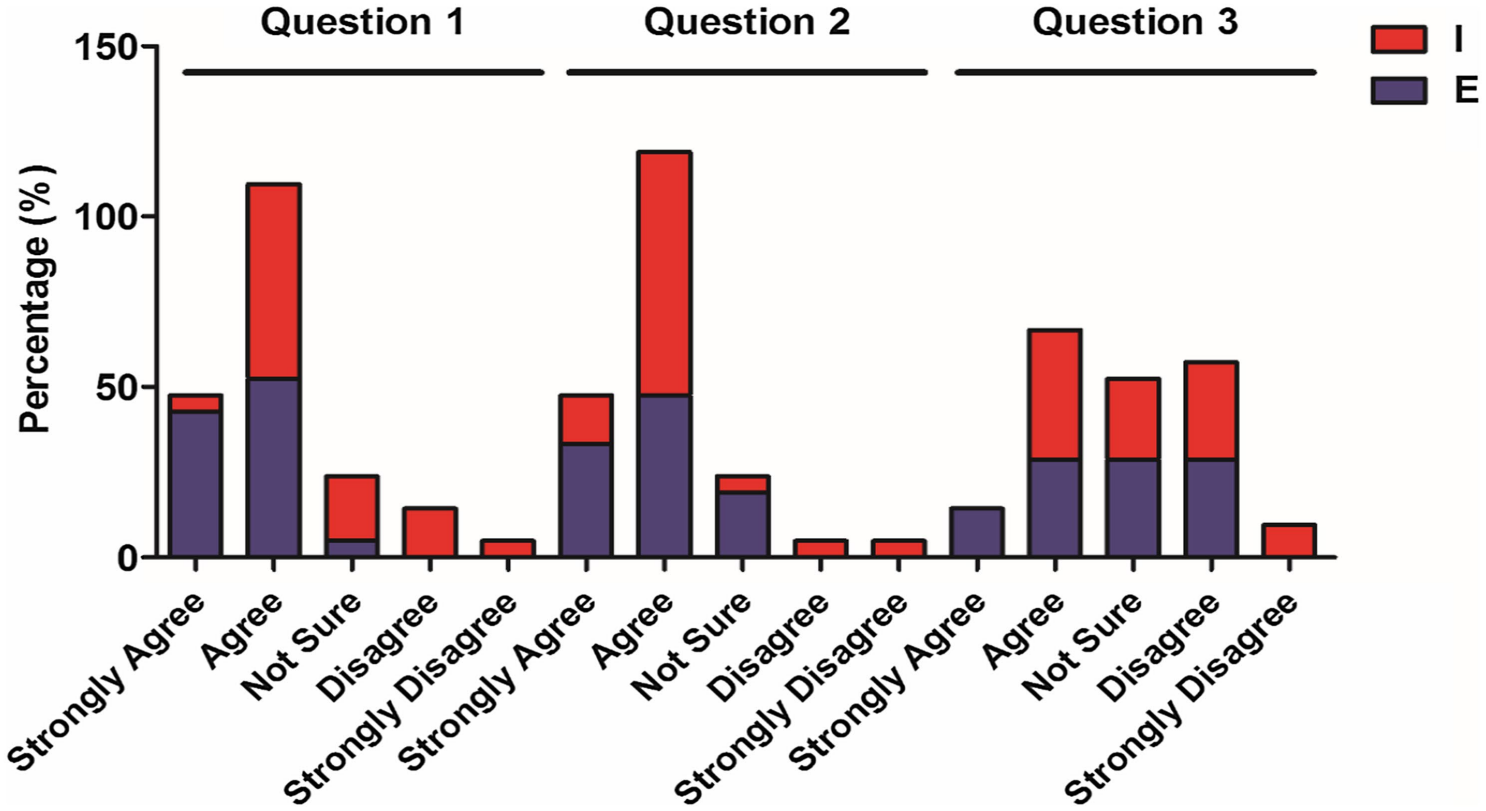
Percentage of elementary and intermediate students’ choices for questions 1–3.

Table 2 and Figure 1 show the responses to questions 1–3 which pertains to students’ general perception of teachers’ code-switching by the frequency and usefulness of code-switching in class. To the first question, which stated that “teachers should sometimes use English while teaching,” the agreement from both levels exceeds 50% (95.3% for the elementary level and 61.9% for the intermediate level). However, analysis showed that a significant percentage of the elementary-level students “strongly agreed” or “agreed” with the statement in question 1, while the intermediate-level students expressed more agreement (57.1%) than strong agreement (4.8%) with the statement. Significantly, while none of the elementary students disagreed that teachers should sometimes use code-switching in class, over 15% of the higher-level students expressed disagreement with this statement.

**Table 2:** Percentage of elementary and intermediate students’ choices for questions 1–3.

Items	Strongly agree (%)		Agree (%)		Not sure (%)		Disagree (%)		Strongly disagree (%)	
	*E*	*I*	*E*	*I*	*E*	*I*	*E*	*I*	*E*	*I*
Q 1	42.9	4.80	52.4	57.1	4.80	19.0	0	14.3	0	4.80
Q 2	33.3	14.3	47.6	71.4	19.0	4.80	0	4.80	0	4.80
Q 3	14.3	0	28.6	28.6	28.6	23.8	28.6	28.6	0	9.50

*E: elementary*. *I: intermediate*. *Q: Question*.

**Figure 2:**
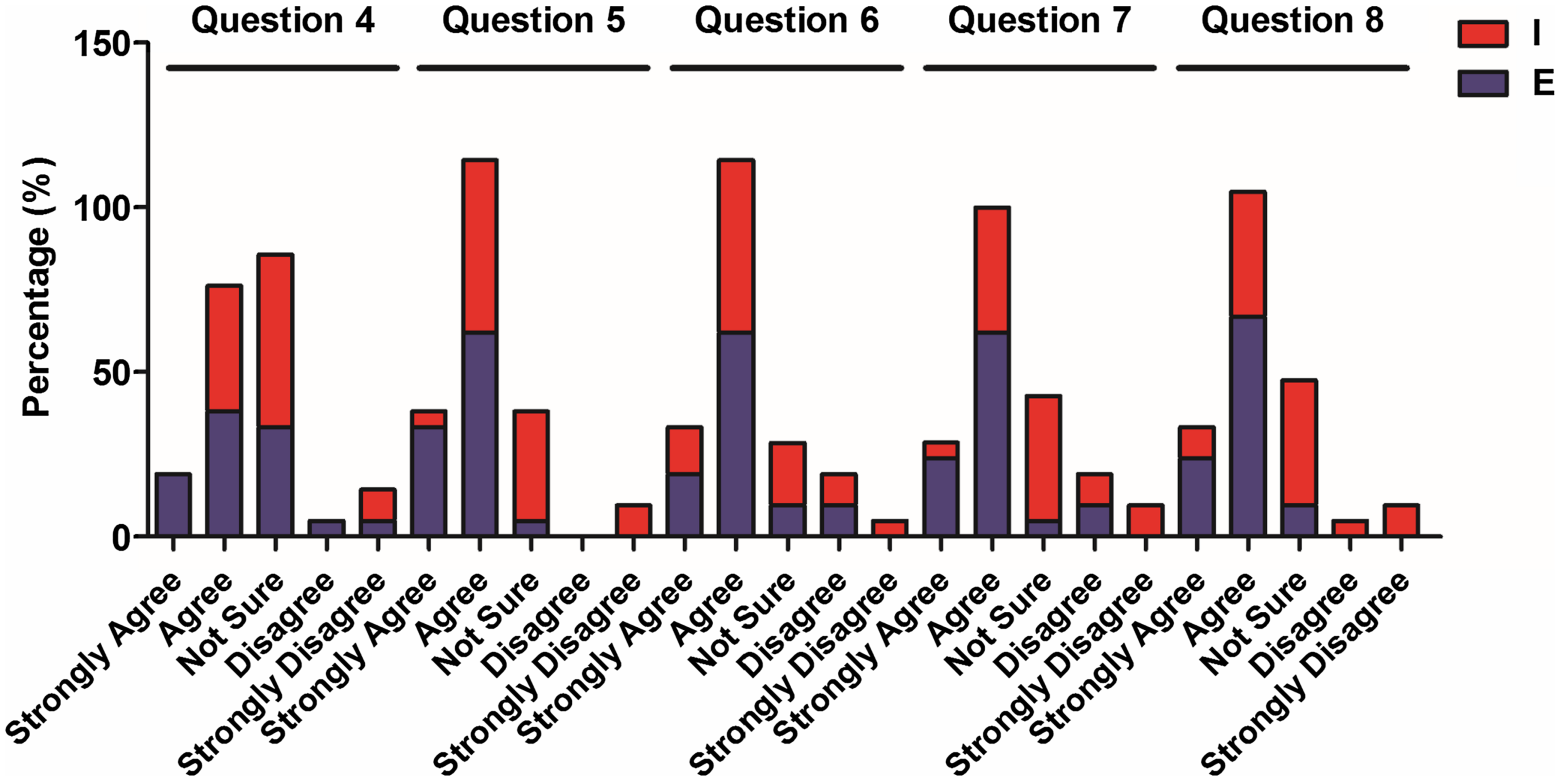
Percentage of elementary and intermediate students’ choices for questions 4–8.

Responses to Question 2 also reveal a high level of agreement from elementary and intermediate students in regard to the helpfulness of teachers’ usage of English to their Chinese learning process (80.9 and 85.7%, respectively). However, the number of the elementary students choosing either “strongly agree” or “agree” was still quite balanced, although significantly more intermediate-level students expressed agreement rather than strong agreement with the statement. The last question in this section pertained to whether teachers should increase the usage of code-switching in class. Surprisingly, the percentages of elementary and intermediate students who selected each option on the scale (general agreement, not sure, and widespread disagreement) were quite similar, ranging between 30 and 40%. Generally speaking, both elementary and intermediate students tended to think that the use of code-switching was helpful to their learning. However, the degree of agreement was higher among those students with a comparably lower level. Overall, most students seemed to expect teachers’ occasional use of code-switching in class, but they did not feel a strong need to increase that frequency. This was also evident from their open-ended answers, wherein both elementary and intermediate students expressed their positive feeling about code-switching, although they did not think it should be overly used in class since they wanted to learn and use more Chinese language (Figure 2).

**Figure 3:**
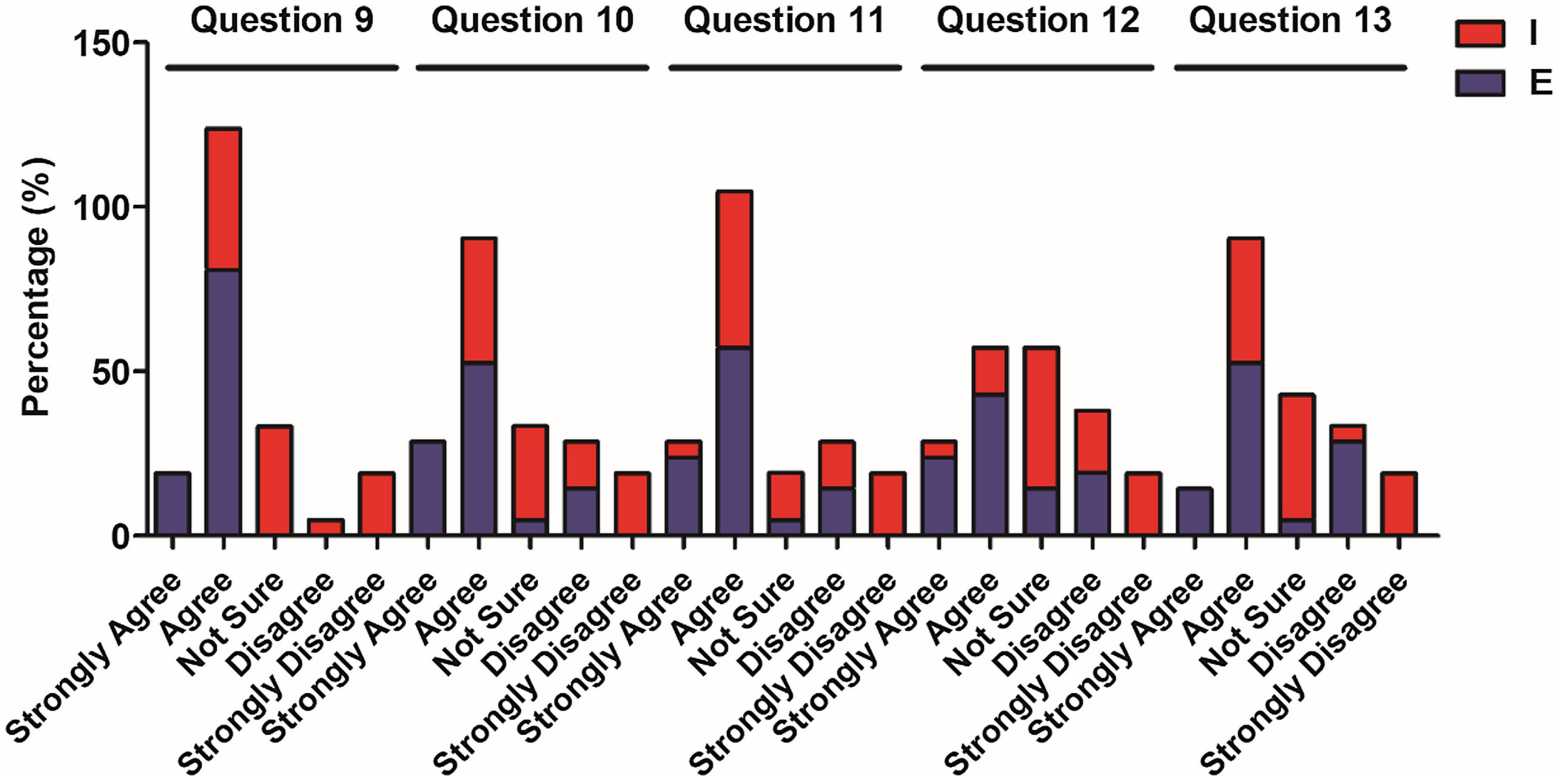
Percentage of elementary and intermediate students’ choices for questions 9–13.

Additionally, in relation to the students’ attitudes on code-switching in the function area of subject access (see Table 3), 57.1% of the elementary students thought that teachers could deploy code-switching for all kinds of topics in class (Question 4). However, 33.3% of the participants were not sure of this, and nearly 10% of them did not agree with the statement. More than 38% of the intermediate students expressed overall agreement, while over 50% of them were uncertain as to this issue. Questioned as to the usefulness of code-switching in explaining grammatical and lexical points (Question 5), over 90% (33.3% “strongly agree” and 61.9% “agree”) of the elementary students expressed agreement with this opinion, while more than 50% (57.2%) of the higher-level students also expressed agreement, although with a comparatively much lower rate. While none of the elementary students expressed disagreement, a small percentage (10%) strongly disagreed as to the helpfulness of this function of code-switching. Responses to Question 6 indicated that over 80 and 65% of the elementary and intermediate levels, respectively, agreed that teachers who alternate codes could help with the explanation of cultural topics. However, nearly 15% of the higher-level students expressed their disagreement over this item. Similar distribution was presented in Question 8 (better clarification of lesson content), except significantly more elementary students (around 90%) a lesser percentage of the intermediate students (47.6%) expressed agreement with it. The analysis of open-ended responses revealed a high occurrence of the opinions that the usage of English could clearly “explain difficult concepts” or help them “understand more” (Figure 3).

**Table 3:** Percentage of elementary and intermediate students’ choices for questions 4–8.

Items	Strongly agree (%)		Agree (%)		Not sure (%)		Disagree (%)		Strongly disagree (%)	
	*E*	*I*	*E*	*I*	*E*	*I*	*E*	*I*	*E*	*I*
Q 4	19.0	0	38.1	38.1	33.3	52.4	4.80	0	4.80	9.50
Q 5	33.3	4.80	61.9	52.4	4.80	33.3	0	0	0	9.50
Q 6	19.0	14.3	61.9	52.4	9.50	19.0	9.50	9.5	0	4.80
Q 7	23.8	4.80	61.9	38.1	4.8	38.1	9.50	9.50	0	9.50
Q 8	23.8	9.50	66.7	38.1	9.50	38.1	0	4.80	0	9.50

**Figure 4:**
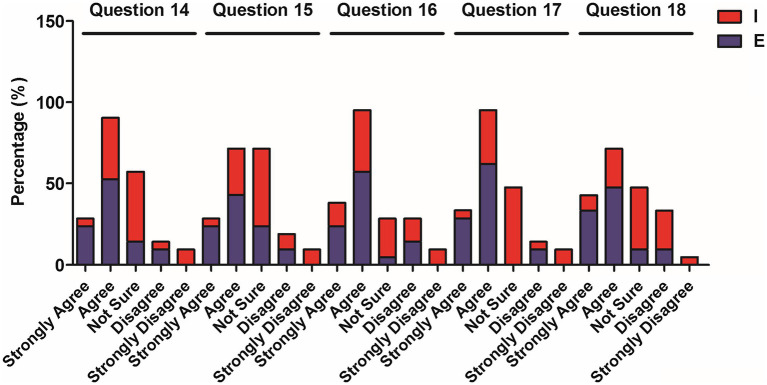
Percentage of elementary and intermediate students’ choices for questions 14–18.

Table 4 presents the results for the function of classroom management of code-switching which shows that the majority of the elementary-level students agree to varying degrees (80.9% chose “agree”; 19% “strongly agree”) that teachers’ code-switching helps them to better understand task instructions. The intermediate students expressed agreement (42.9%) while a lesser percentage (around 24%) agreed with the statement. The remaining percentage of students chose “not sure” in response to the question. Responses to questions 10 (disciplining students) and 11 (engaging students’ attention) reflected a similar distribution of the percentages. Almost 80% of the lower-level students agreed with the statements, while 15% of them disagreed with the statements. On the other hand, 40–50% of the higher-level students agreed with the statements, while 30% of them disagreed with the statements. In terms of code-switching as a technique to keep students quiet (Question 12), almost 70% of the elementary students agreed with the statement, whereas a far lesser percentage of intermediate students (19.1%) expressed agreement with the statement, and only 20% of them disagreed with the statement. A substantial percentage (66.7%) of the elementary-level students agreed that teachers could better call on students, while 38.1% of intermediate-level students expressed agreement with the statement (Question 13). In terms of questions 12 and 13, almost 25% of the elementary and intermediate students were not certain as to the statements (Figure 4).

**Table 4:** Percentage of elementary and intermediate students’ choices for questions 9–13.

Items	Strongly agree (%)		Agree (%)		Not sure (%)		Disagree (%)		Strongly disagree (%)	
	*E*	*I*	*E*	*I*	*E*	*I*	*E*	*I*	*E*	*I*
Q 9	19.0	0	80.9	42.9	0	33.3	0	4.80	0	19.0
Q 10	28.6	0	52.4	38.1	4.80	28.6	14.3	14.3	0	19.0
Q 11	23.8	4.80	57.1	47.6	4.80	14.3	14.3	14.3	0	19.0
Q 12	23.8	4.80	42.9	14.3	14.3	42.9	19.0	19.1	0	19.0
Q 13	14.3	0	52.4	38.1	4.8	38.1	28.6	4.80	0	19.0

Table 5 showcases the results from the responses to questions 14–18 and exhibits the function area of interpersonal relations built between teachers and students (Table 1). In response to questions 14 and 15 (code-switching can better encourage and praise students), nearly 70% of the elementary students expressed agreement, with the statement, whereas around 15–19% of the intermediate students expressed disagreement. Responding to question 16, more than 80 and 50% of the elementary and intermediate students agreed that code-switching helped to relax the class atmosphere. In terms of the functions of commenting on students’ response and reducing distance (question 17), 90.5% of the elementary students agreed with the statement, while only 38.1% of the higher-level learners agreed with it. Similarly, in their responses to question 18, a greater percentage (more than 80% of the lower-level students) expressed agreement, while only 30% of intermediate students showed agreement. Analysis of data demonstrated a stark contrast in terms of the degrees of agreement to both questions by students from the two proficiency levels. In the open-ended question, some students from the lower level also expressed their positive attitudes toward the interpersonal function of code-switching, commenting that “[it helps] and for asking I am too shy so I could not ask. English can help me understand jokes and grammar, etc.”

**Table 5:** Percentage of elementary and intermediate students’ choices for questions 14–18.

Items	Strongly agree (%)		Agree (%)		Not sure (%)		Disagree (%)		Strongly disagree (%)	
	*E*	*I*	*E*	*I*	*E*	*I*	*E*	*I*	*E*	*I*
Q 14	23.8	4.80	52.4	38.1	14.3	42.9	9.50	4.80	0	9.50
Q 15	23.8	4.80	42.9	28.6	23.8	47.6	9.50	9.5	0	9.50
Q 16	23.8	14.3	57.1	38.1	4.8	23.8	14.3	14.3	0	9.50
Q 17	28.6	4.8	61.9	33.3	0	47.6	9.50	4.8	0	9.50
Q 18	33.3	9.5	47.6	23.8	9.50	38.1	9.50	23.8	0	4.80

In order to ascertain if the perceptions from the two proficiency levels differed significantly and in which functions they differed significantly, the Mann–Whitney U test was conducted. As the results show Table 6, Questions 1, 5, 7, 8, 10, 11, 12, 14, 15, 17, and 18 were demonstrated to be significantly different since their *p*-values were 0.001, 0.001, 0.005, 0.004, 0.002, 0.018, 0.004, 0.006, 0.022, 0.023, 0.001, and 0.004, respectively (*p* < 0.05). In question 1, as mentioned in the last section, the agreement of the elementary-level students was significantly higher than that of the intermediate-level students. In question 5, although the data showed that the opinions across the two levels differed significantly, an overall positive perception (more than 50% agreement) toward this specific function was in evidence. Questions 7, 8, 10, 11, and 12 attracted similar responses in that more than 80% of the elementary students expressed agreement or strong agreement with each statement, although only 20–30% of the higher-level students provided the same kind of positive answers. In questions 12, 14, 15, 17, and 18, the students’ choices not only differed significantly in terms of their agreement toward the items, but also in terms of their choice of the uncertainty (e.g., in Question 17, 47.6% of the intermediate students chose “not sure” while no one chose this option in the elementary level). It can be seen from the U test that lower-level students showed a decided preference code-switching. On the other hand, the higher-level students exhibited considerable uncertainty toward this strategy.

**Table 6:** Result of Mann–Whitney U test.

Items	Mann–Whitney U	Wilcoxon W	*Z*	Asymp Sig (2-tailed)
Q 1	96.500	327.500	−3.443	0.001^*^
Q 2	189.500	420.500	−0.886	0.376
Q 3	183.000	414.000	−0.983	0.326
Q 4	161.500	392.500	−1.487	0.137
Q 5	91.500	322.500	−3.291	0.001^*^
Q 6	193.500	424.500	−0.953	0.341
Q 7	121.000	352.000	−2.818	0.005^*^
Q 8	112.000	343.000	−2.843	0.004^*^
Q 9	76.500	307.500	−4.164	0.000
Q 10	109.500	340.500	−3.161	0.002^*^
Q 11	139.000	370.000	−2.375	0.018^*^
Q 12	114.500	345.500	−2.849	0.004^*^
Q 13	151.000	382.000	−1.744	0.081
Q 14	135.000	366.000	−2.291	0.022^*^
Q 15	134.500	365.500	−2.273	0.023^*^
Q 16	163.000	394.000	−1.690	0.091
Q 17	98.500	329.500	−3.283	0.001^*^
Q 18	109.000	340.000	−2.913	0.004^*^

* *p < 0.05 (asterisk indicates significance)*.

In the questionnaires collected from the participants, 24 of the sheets were valid in terms of the open-ended questions, while in the remaining questionnaires, the open-ended question was not answered. Analysis of all valid responses showed that 16 of the students were in favor of using English as a code-switching strategy in the CFL classroom, while 8 of them express doubts about the strategy. Of the ones who are in support of the code-switching, they found several aspects of this strategy helpful for slowing down the class pace (S1: “Good, because sometimes teacher speaks very quickly so I cannot catch what she says”) and for facilitating their understandings of class contents (S2: “Good, I can understand better”; moreover, teacher’s switching to English eases their understanding problems especially when difficult concepts and grammars are explained). S16 noted that it was “Not so bad, because when we study grammar, then English is okay but other situation Chinese is more better,” while S23 commented that “I feel English should be used occasionally for teachers to explain difficult concepts, especially for students with poor Chinese background.” It was also found that teacher’s use of English helped to build rapport between the teacher and students. For instance, S14 commented that it was “of course good! Because sometimes I do not understand Chinese words or something else. And for asking I’m too shy, so I cannot ask. English can help to understand jokes, grammar, etc..” According to her response, she feels more comfortable when the teacher’s jokes and her own questions are delivered in English.

In terms of the students who were not in favor of English as the code-switching medium, they felt that the target language (in this case, the Chinese language) should be used more frequently in a CFL classroom. For example, S38 noted that “Because this is Chinese class so teachers should use Chinese instead of using English.” S15 elaborated that “It’s okay if they use English sometimes, but I feel the use of a lot of English is bad. [Using English] is fine only in explaining the words in English to better understand.” Further some of the students expressed a negative attitude toward English as the code-switching medium for they had limited understanding of the English language. However, as mentioned in section “Research Instrument**”**, among the international students learning Chinese at CCLC, 80% of them spoke English as their first language, while 20% of them did not speak English as a first language. As a result, instead of supporting and facilitating their classroom understanding, the teacher’s English code-switching strategy added to the stress at their lack of comprehension. For example, 3 of the 8 students who did not support code-switching observed that.

*not all of the foreign students can understand English and besides, this is Chinese class so better use Chinese (S:37).*

我觉得老是用英文，我们的进步很慢。还有我不会说英语 *[literal translation: I think if the teacher always uses English, we will progress slowly. And I do not speak English] (S:19).*

*No, because sometimes students dos not understand that teachers are saying* (*S:17).*

## Discussion

In this paper, we studied the perceptions of elementary and intermediate CFL learners toward teachers’ code-switching strategy. For this purpose, we collected survey data, including responses to closed-ended questions and open-ended questions from the students enrolled in the College of Chinese Language and Culture (CCLC) of Jinan University, where CFL classes are offered to international students. In our study, questionnaire data demonstrated (Table 2; Figure 1) confirmed the findings from earlier studies by (Wang, 2010; Wang and Kirkpatrick, 2012) that incorporating English as a code-switching strategy is overall a powerful pedagogical tool in CFL classrooms. However, the comparison of the perceptions of learners across different proficiency levels using the Mann–Whitney test suggests that learners with lower proficiency levels tend to favor teacher code-switching more than the higher-level learners. This is in line with studies carried out by Xu (2008), Ahmad and Jusoff (2009), Yao (2011) and Rezaee and Fathi (2017). It seems code-switching is generally supported more by lower-level students in different language teaching contexts.

The analysis of Tables 3–5 (the questionnaire data) and Table 6 (the Mann–Whitney U test data) reveals CFL learners’ attitudes toward the different functions of code-switching. In terms of subject access, the functions of explaining grammatical points and lexical items, evoking responses, and clarifying content were revealed to benefit lower-level students more than higher-level ones, while both levels expressed a positive response to situations in which the teachers switched to their L1 to elaborate cultural topics.

Across the three functions, the learners’ opinions differed more significantly in terms of the functions of classroom management and interpersonal relations. Higher-level learners tended to agree less as to this function which aligns with the findings of the study by Zhang et al. (2020)’s study suggesting that teachers at CFL classrooms provide classroom instructions in Chinese so that a Chinese-only environment is created. Meanwhile, compared to elementary-level learners, intermediate-level learners tended to agree less and express more doubts on the interpersonal functions of code-switching. This result diverges from the findings of previous studies in the EFL context (e.g., Ahmad and Jusoff, 2009; Nordin et al., 2013) which found support for the positive role of the code-switching function of building affect in classrooms. The findings in the current study may be attributed to the teacher-centered norm and low interaction frequency between the teacher and learners in Chinese classrooms (Malik et al., 2017).

So far, the findings in the context of the present study are similar to those of the previous EFL studies, thus confirming their findings. Where the findings of the current study differ is in the considerable variation in perceptions across the two levels (in that nearly half of the intermediate learners expressed uncertainty) in terms of keeping the students quiet, encouraging and praising students, commenting on students, and reducing teacher-student distance. In other studies, students rarely chose “not sure” (e.g., Yao, 2011). The high frequency of students choosing “not sure” under the category of interpersonal relations in this study might be due to the lack of occurrences of code-switching and teacher-student interaction in the intermediate class, in which there is greater focus on adequate teaching of content and faster teaching pace. This is in line with Wang’s (2010) interpretation of the CFL students she observed that “It implies students might have never heard their teachers speaking English or felt hard to evaluate” (p. 258). Moreover, this is also in line with the “teacher-centeredness” norm of Chinese teachers revealed by Wang and Kirkpatrick (2012). The more frequent selection of the “not sure” option might also be due to the fact that a certain number of the students do not speak English as their L1. In this case, using English as the medium of code-switching does not help their understanding in class.

## Conclusion

The current paper is based on a quantitative and qualitative examination of different proficiency-level learners’ perceptions of code-switching as a teaching strategy in the CFL context. The data suggest that code-switching is an effective tool to aid students in learning Chinese as their L2. Comparably, students at the elementary level are more in favor of code-switching than those studying CFL at the higher level.

Based on analysis of the data, a number of pedagogical recommendations are offered. Firstly, teachers should pay attention to the scaffolding strategy when applying code-switching. In the context of the lower-level students, more code-switching can be used to help them familiarize themselves with the Chinese language. In the context of advanced students, the teachers should deploy code-switching to facilitate students’ development. In terms of the specific functions of code-switching, it is suggested that teachers can use code-switching for explanation more frequently when students are introduced to new cultural topics and concepts. In term of classroom management, while intermediate students, teachers are advised to use code-switching less. They are suggested to consciously instruct and manage the class in Chinese. Conventional and repetitive phrases to manage class and praise students in Chinese can be used more often to create a more Chinese-immersive classroom. It is also suggested that teachers should use code-switching more frequently for the purpose of building affect with students, since students perceived this more positively. Last, it should be acknowledged that not all international CFL learners speak English as their native language. Consequently, using English as the medium to code-switch may sometimes confuse such students. The solution to this problem can be investigated in future studies.

## Data Availability Statement

The raw data supporting the conclusions of this article will be made available by the authors, without undue reservation.

## Author Contributions

YH has drafted the paper’s literature review and did the analysis. MA has contributed in writing the introduction and overall editing of the manuscript. However, HA has participated in the writing discussion section of the paper. All authors contributed to the article and approved the submitted version.

## Conflict of Interest

The authors declare that the research was conducted in the absence of any commercial or financial relationships that could be construed as a potential conflict of interest.

## Publisher’s Note

All claims expressed in this article are solely those of the authors and do not necessarily represent those of their affiliated organizations, or those of the publisher, the editors and the reviewers. Any product that may be evaluated in this article, or claim that may be made by its manufacturer, is not guaranteed or endorsed by the publisher.

## Footnotes

^1^ https://zsb.jnu.edu.cn/b6/4f/c4284a112207/pagem.psp
